# Osthole Ameliorates Renal Fibrosis in Mice by Suppressing Fibroblast Activation and Epithelial-Mesenchymal Transition

**DOI:** 10.3389/fphys.2018.01650

**Published:** 2018-11-21

**Authors:** Suping Zhang, Qian Huang, Xiaoxia Cai, Shan Jiang, Nan Xu, Qin Zhou, Xiaoyun Cao, Michael Hultström, Jiong Tian, En Yin Lai

**Affiliations:** ^1^Kidney Disease Center, First Affiliated Hospital, College of Medicine, Zhejiang University, Hangzhou, China; ^2^Department of Physiology, School of Basic Medical Sciences, Zhejiang University School of Medicine, Hangzhou, China; ^3^Department of Physiology, Quanzhou Medical College, Quanzhou, China; ^4^Department of Basic Medical Sciences, Honghe Health Vocational College, Mengzi, China; ^5^Integrative Physiology, Department of Medical Cell Biology, Uppsala University, Uppsala, Sweden; ^6^Anaesthesia and Intensive Care Medicine, Department of Surgical Sciences, Uppsala University, Uppsala, Sweden

**Keywords:** osthole, fibroblast, EMT, inflammation, renal fibrosis

## Abstract

Renal fibrosis is a common pathway of virtually all progressive kidney diseases. Osthole (OST, 7-Methoxy-8-(3-methylbut-2-enyl)-2-chromenone), a derivative of coumarin mainly found in plants of the *Apiaceae* family, has shown inhibitory effects on inflammation, oxidative stress, fibrosis and tumor progression. The present study investigated whether OST mediates its effect via suppressing fibroblast activation and epithelial-mesenchymal transition (EMT) in unilateral ureteral obstruction (UUO)-induced renal fibrosis in mice. Herein, we found that OST inhibited fibroblast activation in a dose-dependent manner by inhibiting the transforming growth factor-β1 (TGFβ1)-Smad pathway. OST also blocked fibroblast proliferation by reducing DNA synthesis and downregulating the expressions of proliferation- and cell cycle-related proteins including proliferating cell nuclear antigen (PCNA), CyclinD1 and p21 Waf1/Cip1. Meanwhile, in the murine model of renal interstitial fibrosis induced by UUO, myofibroblast activation with increased expression of α-smooth muscle actin (α-SMA) and proliferation were attenuated by OST treatment. Additionally, we provided *in vivo* evidence suggesting that OST repressed EMT with preserved E-cadherin and reduced Vimentin expression in obstructed kidney. UUO injury-induced upregulation of EMT-related transcription factors, Snail family transcriptional repressor-1(Snail 1) and Twist family basic helix-loop-helix (BHLH) transcription factor (Twist) as well as elevated G2/M arrest of tubular epithelial cell, were rescued by OST treatment. Further, OST treatment reversed aberrant expression of TGFβ1-Smad signaling pathway, increased level of proinflammatory cytokines and NF-kappaB (NF-κB) activation in kidneys with obstructive nephropathy. Taken together, these findings suggest that OST hinder renal fibrosis in UUO mouse mainly through inhibition of fibroblast activation and EMT.

## Introduction

Renal fibrosis caused by deposition and accumulation of extracellular matrix (ECM) components is the common final pathway of chronic kidney disease. Multiple cellular events are involved in this process, including fibroblast activation, epithelial to mesenchymal transition (EMT), inflammatory cells infiltration, and tubular epithelial cell apoptosis (Zeisberg and Neilson, 2010; Liu, 2011). Currently, there are no generally effective treatments for preventing the progression of renal fibrosis (Nogueira et al., 2017).

Osthole (OST, 7-Methoxy-8-(3-methylbut-2-enyl)-2-chromenone) is a derivative of coumarin mainly found in plants of the *Apiaceae* family, *Cnidium monnieri* and *Angelica pubescens*, which are commonly used in traditional Chinese medicine (You et al., 2009; Zhang Z.R. et al., 2015). The pharmacological mechanism of its action is still under investigation, but several potentially therapeutic effects have been demonstrated. It has been found to reduce tumor progression (Lin et al., 2014; Feng et al., 2017), have antibiotic properties (Wang et al., 2009), reduce allergic reactions (Chiang et al., 2017) and ameliorate osteoporosis (Zhang et al., 2017). Previous studies have indicated that OST may reduce fibrosis in the lung, liver and heart (Chen et al., 2011; Liu et al., 2015; Hao and Liu, 2016), but in renal tissue it is less studied. Thus, the present investigation was to focus on the effect of OST on renal tissue.

Fibrogenesis is the deposition of pathological matrix. It is widely recognized that local fibroblast activation featured with proliferation and phenotypic appearance of myofibroblasts (α-SMA^+^ fibroblasts) mainly contributes to inappropriate matrix expansion and consequent renal structural deformations (Barnes and Gorin, 2011; Liu, 2011; LeBleu et al., 2013). Partial EMT is a feature of kidney fibrosis with tubular epithelial cells acquiring mesenchymal cell marker but the preserved integrity of the renal tubules (Grande et al., 2015). EMT program of renal epithelial cells and consequent G2 cell-cycle arrest promote kidney fibrosis through an altered secretome profile, leading to the accumulation of interstitial myofibroblasts and the compromised kidney parenchyma function (Lovisa et al., 2015; Djudjaj et al., 2017; Gewin, 2018). Transforming growth factor -beta1 (TGF-β1) is one of the most important profibrotic cytokines in chronic kidney disease (Meng et al., 2016), and exerts its effects mainly through the Smad pathway (Roberts et al., 1986; Desmouliere et al., 1993). Upon TGF-β1 stimulation, Smad2 and Smad3 are phosphorylated and activated, then form complex with Smad4 and translocate to the nucleus where they function as transcription factors for many fibrosis associated genes (Miyazono et al., 2000; Verrecchia et al., 2001). Activation of Smad3, especially in tubular epithelial cells or fibroblasts, is clearly involved in renal tubulointerstitial fibrosis through enhancing of EMT program, inflammatory cells influx and collagen accumulation (Sato et al., 2003; Meng et al., 2010). Smad7, an intracellular antagonist for TGF-β signaling, is degraded in progression of tubulointerstitial fibrosis (Fukasawa et al., 2004). Dysregulation of Smad7 promotes renal fibrosis (Chung et al., 2013) and conversely Smad7 treatment substantially attenuating progressive kidney diseases via inactivating TGF-β/Smad3 signaling (Zhou et al., 2016). Profibrotic cytokines secreted from inflammatory cells and injured tubular cells build up an inflammatory state in the kidney, arguably providing a directional or indirectional signal to trigger activation of resident fibroblasts and phenotypic transition of tubular cells (Liu, 2011). NF-kappaB (NF-κB), the major inflammatory response pathway (Blackwell and Christman, 1997), generates a loop that maintains the inflammatory signals is also the driving force for renal fibrosis (Miyajima et al., 2003; Tan et al., 2008). Indeed, increasing the levels of the endogenous inhibitor of NF-κB, I-κB, or inhibiting the pathway diminishes the expression of inflammatory genes, inhibits EMT phenotypes and decreases deposition of interstitial ECM (Inoue et al., 2010; Li et al., 2011).

The aim of this study was to assess the effect of OST on renal fibrosis in the mouse model of renal interstitial fibrosis induced by unilateral ureteral obstruction (UUO) and to examine if it could work through inhibiting renal fibroblast proliferation and EMT program. The main signaling pathways that involved in renal fibro-genesis such as TGF-β/Smad and the NFκB pathway were also investigated.

## Materials and Methods

### Animals and Experimental Model

Adult male C57Bl/6 mice weighing 20–25 g were obtained from Shanghai Laboratory Animal Center (Shanghai, China) and housed in a temperature and humidity-controlled environment with free access to food and water. All animal experiments were conducted with approval from the Institute Animal Care and Ethical Committee of Zhejiang University School of Medicine. OST (MedChem Express, NJ, United States) was prepared in 0.5% sodium carboxymethyl cellulose (CMC) solution. The mouse was given OST once a day intragastrically starting from the day before the UUO surgery for 1 week. Sham and UUO mice received the equal amount of DMSO in carrier solution. Sham and UUO mice received the equal amount of DMSO in carrier solution. Mice were randomly divided into five groups: Sham, Sham + OST (80 mg/kg/day, i.g.), UUO, UUO + OST (40 mg/kg/day, i.g.) and UUO + OST (80 mg/kg/day, i.g.). UUO was performed as previously described (Chevalier et al., 2009; Tveitaras et al., 2015). Briefly, surgery was performed under isoflurane anesthesia. The left ureter was visualized following a flank incision and ligated using a 4.0 silk suture at two points along its length (UUO-operated animals) or similarly manipulated but not ligated (sham-operated animals). Mice were sacrificed on day 7, and obstructed kidneys were harvested for pathological and molecular analyses.

### Morphological and Immunohistochemical Analyses

Kidney samples were fixed in 4% Paraformaldehyde (PFA), embedded in paraffin, and sectioned 4 μm thick for histological analysis. Masson trichrome (MTC) staining was performed to assess tissue fibrotic changes. Ten randomly selected fields (200× magnifications) from each section were analyzed by Image J (National Institutes of Health, Bethesda, MD, United States). The severity of tubulointerstitial fibrosis was indicated as the ratio of blue-stained scarred areas to the total area. For immunohistochemical studies, renal sections were incubated with antibodies against α-SMA (ab5694, Abcam, United Kingdom), Fibronectin (FN, ab45688, Abcam, United Kingdom), Collagen I (Col I, ab34710, Abcam, United States), E-cadherin (#3195, Cell Signaling Technology, United States), Vimentin (ab92547, Cell Signaling Technology, United States), Phospho-NF-κB p65 (Ser536, ab86299, Abcam, United Kingdom), Phospho-histone H3 (Ser10, #9701, Cell Signaling Technology, United States) and Phospho-smad3 (Abcam, ab52903, United Kingdom). After biotinylated secondary antibody was applied, the slides were detected by the DAB Horseradish Peroxidase Color Development Kit (Beyotime, Shanghai, China).

### Real-Time RT-PCR

Total RNA was isolated from frozen kidney tissues using Trizol (Invitrogen, Waltham, MA, United States) and then reversely transcribed to cDNA with the PrimeScript^TM^ RT reagent Kit (TaKaRa Biotech, Shiga, Japan). For quantitative PCR, assays were performed in triplicate on an Applied Biosystems 7500 Fast Real-Time PCR System using SYBRGreen Master Mix and gene-specific primers listed in Table 1. The mRNA levels were normalized by GAPDH expression in the same sample.

**Table 1:** Sequences of primers for quantitative PCR.

Primer name	Primer sequence
ICAM-1_fw	CGCAGAGGACCTTAACAGTCTACAAC
ICAM-1_rev	GACGCCGCTCAGAAGAACCAC
TNF-α_fw	GCGACGTGGAACTGGCAGAAG
TNF-α_rev	GAATGAGAAGAGGCTGAGACATAGGC
MCP-1_fw	CCACTCACCTGCTGCTACTCATTC
MCP-1_rev	CTGCTGCTGGTGATCCTCTTGTAG
IL-6_fw	AGACTTCCATCCAGTTGCCTTCTTG
IL-6_rev	CATGTGTAATTAAGCCTCCGACTTGTG
IL-1β_fw	TTCAGGCAGGCAGTATCACTCATTG
IL-1β_rev	ACACCAGCAGGTTATCATCATCATCC
GAPDH_fw	TCACCATCTTCCAGGAGCGAGAC
GAPDH_rev	AGACACCAGTAGACTCCACGACATAC

### Cell Culture and Treatment

Rat kidney interstitial fibroblasts (NRK-49F cells) were obtained from American Type Culture Collection (ATCC, CRL-1570TM) and cultured at 37°C, 5% CO_2_ in DMEM/F-12 medium (GIBCO, NY, United States) supplemented with 10% fetal bovine serum. Recombinant human TGF-β1 (R&D systems) was used to stimulate cells *in vitro*. Briefly, cells were seeded onto six-well culture plates to 60–70% confluence in complete medium containing 10% fetal bovine serum and then changed to serum-free medium for 24 h. After pre-incubation with OST at various concentrations (10–40 μM) for 30 min, TGF-β1 (5 ng/ml) was added for an additional 12 or 24 h before harvesting. OST used in cellular experiments was dissolved in 0.1% DMSO (Sigma-Aldrich). And cells incubated with an equivalent amount of DMSO were used as control.

### Cell Proliferation Assay

Cell proliferation was assessed by MTT assay and EdU incorporation. For the MTT assay, cells (2 × 10^3^/100 ml) were seeded in 96-well plate. After starved with serum free medium for 24 h, cells were pre-treated with OST at various concentrations for 30 min, followed by incubation with or without 10% fetal bovine serum (FBS) for 24 h. MTT (5 mg/ml) was added at 4 h before treatment termination (Pang et al., 2011; Song K. et al., 2014). Then the medium was removed and DMSO was added to dissolve the formazan crystals. Absorbance of each well was measured by a microplate reader at 490 nm. For the EdU incorporation, a Click-iT EdU Alexa 488 Imaging kit (Beyotime, Shanghai, China) was used according to the manufacturer’s instructions.

### Immunofluorescence Staining

Paraffin-embedded kidney sections were deparaffinized, hydrated and subjected to antigen-retrieval. After blocked with 3% BSA in PBS for 1 h, the sections were incubated with primary antibodies against α-SMA, ki-67. Alexa Fluor^®^ 488, Alexa Fluor^®^ 555-conjugated secondary antibodies were used to visualize the primary antibodies. Cells (on cover-slips) with or without various drug treatments for 24 h were fixed with 4% paraformaldehyde, permeabilized with 0.1% Triton X100, blocked with 3% BSA and incubated with the antibody against α-SMA, fibronectin, phosphorylated smad3. Subsequently, the cells were exposed to the Cy3 or Alexa Fluor^®^ 488-conjugated secondary antibodies for 1 h at room temperature. The nuclei were counterstained with DAPI. Fluorescent images were collected with a confocal microscope (Olympus FV3000, Tokyo, Japan).

### Western Blot Analysis

Kidney and cell culture samples were lysed with RIPA buffer containing protease inhibitors cocktail and centrifuged at 12000 g for 5 min. Then supernatants were collected. Protein concentration estimations were determined using the BCA protein assay kit (Solarbio, Beijing, China). Equal amounts of protein were separated by SDS-polyacrylamide gel electrophoresis and transferred onto polyvinylidene difluoride membranes. The membranes were blocked in 5% non-fat dry milk and incubated with specific primary antibodies for α-SMA (ab5694, Abcam, United Kingdom), FN (ab45688, Abcam, United Kingdom), Collagen I (Col I, ab21286, Abcam, United Kingdom), NF-κB p65 (#8242, Cell Signaling Technology, United States), Phospho-NF-κB p65 (Ser536, ab86299, Abcam, United Kingdom), smad3 (ab40854, Abcam, United Kingdom), Phospho-smad3(ab52903, Abcam, United Kingdom), CyclinD1 (ab134175, Abcam, United Kingdom), PCNA (ab18197, Abcam, United Kingdom) and p21 Waf1/Cip1 (ab109199, Abcam, United Kingdom), and Phospho-histone H3 (p-H3, #9701, Cell Signaling Technology, United States), E-cadherin (#3195, Cell Signaling Technology, United States) Vimentin (ab92547, Abcam, United Kingdom), GAPDH (#5174, Cell Signaling Technology, MA, United States), Snail1 (#271977, Santa Cruz, CA, United States) and Twist (ab49254, Abcam, United Kingdom) followed by incubation with the secondary antibody (horseradish peroxidase-labeled IgG anti-rabbit/mouse antibody, Cell Signaling Technology). Bands were visualized by enhanced chemiluminescent substrates (ECL, Thermo Scientific) and analyzed using Image J software (National Institutes of Health, Bethesda, MD, United States).

Data were presented as means ± SEM values. *t*-Test and One-Way ANOVA followed by Student–Newman–Keuls *post hoc* test were used for comparisons between groups. All statistical calculations were made using Graphpad Prism (7.0, La Jolla, CA, United States). *P*-value < 0.05 was accepted as statistically significant.

## Results

### OST Suppresses Activation and Proliferation of Renal Fibroblasts *in vitro*

**FIGURE 1:**
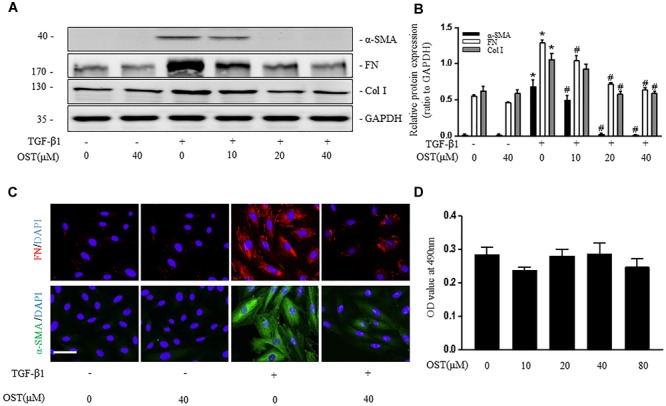
OST suppress activation of NRK-49F cells. NRK-49F were preincubated with OST for 30 min before TGFβ1 (5 ng/ml) and were harvested 24 h after TGFβ1 stimulation. Representative bands **(A)** and Western blot analyses **(B)** of α-SMA, Col I (collagen I) and FN (fibronectin). **(C)** Representative micrographs of immunofluorescent staining of FN (red) and α-SMA (green) (magnification of 400×). Scale bar = 100 μm. **(D)** OST did not affect the viability or proliferation of the cells. NRK 49F Cells were incubated with indicated amount (0–80 μM) of OST for 24h. Cell viability was determined by MTT assay. Data are expressed as mean ± SEM. ^∗^*P* < 0.05 versus untreated cells; ^#^*P* < 0.05 versus TGFβ1- stimulated cells.

After 24 h stimulation with TGF-β1, expression of α-SMA, FN (Fibronectin) and Col (collagen) I were significantly increased in cultured fibroblasts (NRK-49F), whereas this effect was inhibited by administration of OST in a dose-dependent manner (Figures 1A,B). Consistent results were obtained by immunofluorescent staining for α-SMA and FN in NRK-49F (Figure 1C). OST at the concentrations tested in cells did not show considerable cytotoxicity as evidenced by MTT assay (Figure 1D).

**FIGURE 2:**
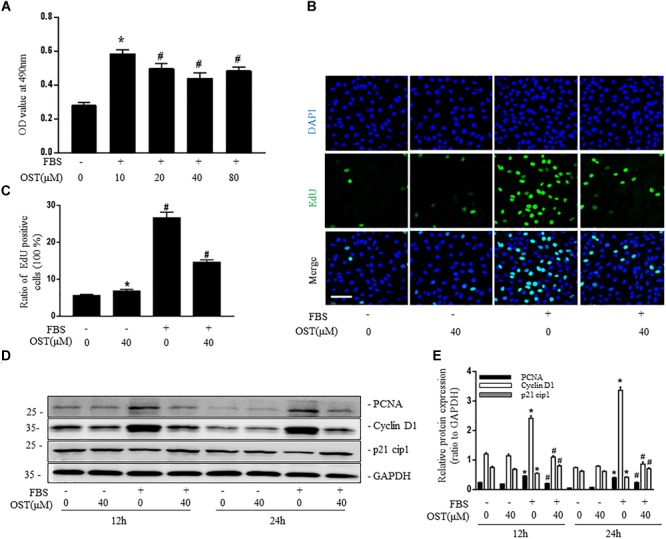
OST suppress proliferation of NRK-49F cells. NRK-49F were preincubated with OST for 30 min before 10% FBS and were harvested 24 h after FBS stimulation. **(A)** MTT and **(B,C)** EdU incorporation assays of different groups (magnification of 200×). Scale bar = 100 μm. Representative bands **(D)** and western blot analyses **(E)** of PCNA, cyclin D1 and p21 cip1. Data are expressed as mean ± SEM. ^∗^*P* < 0.05 versus untreated cells; ^#^*P* < 0.05 versus FBS-stimulated cells.

Pretreatment with OST significantly decreased proliferation of NRK-49F with maximum inhibition at 40 μM (Figure 2A). Quantitative assessment of EdU incorporation also revealed that number of EdU-positive cells in FBS group was reduced from 26.6 to 14.6% after coincubation with OST (40 μM) for 24 h (Figures 2B,C). In addition, NRK-49F were harvested at 12 and 24 h for immunoblot analysis of PCNA (proliferating cell nuclear antigen) and cell cycle proteins (cyclin D1 and p21 Waf1/Cip1). OST at dose of 40 μM prevented FBS-induced up-regulation of PCNA and cyclin D1, which promote progression through the cell cycle, and down-regulation of p21 cip1, a negative regulator of cell cycle (Figures 2D,E).

### OST Inhibit Myofibroblast Activation and Proliferation in UUO-Injured Kidneys

**FIGURE 3:**
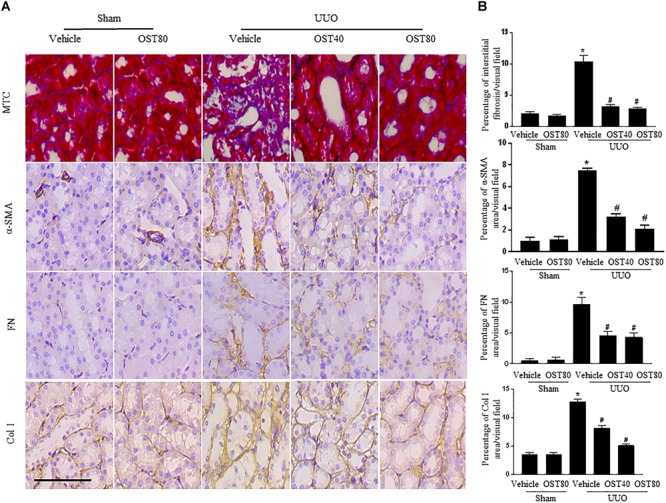
OST ameliorates renal interstitial fibrosis and myofibroblast activation in UUO mice. Mice received daily intragastric administration of vehicle or OST (40 and 80 mg/kg per day) 1 day before UUO and were sacrificed at 1 week after surgery. **(A)** Representative micrographs and quantification **(B)** of Masson’s trichrome staining and protein expression of α-SMA, FN and Col I in the obstructed kidneys from different groups as indicated. Scale bar = 100 μm. Data are expressed as mean ± SEM. ^∗^*P* < 0.05 versus Sham + Vehicle; ^#^*P* < 0.05 versus UUO + Vehicle.

**FIGURE 4:**
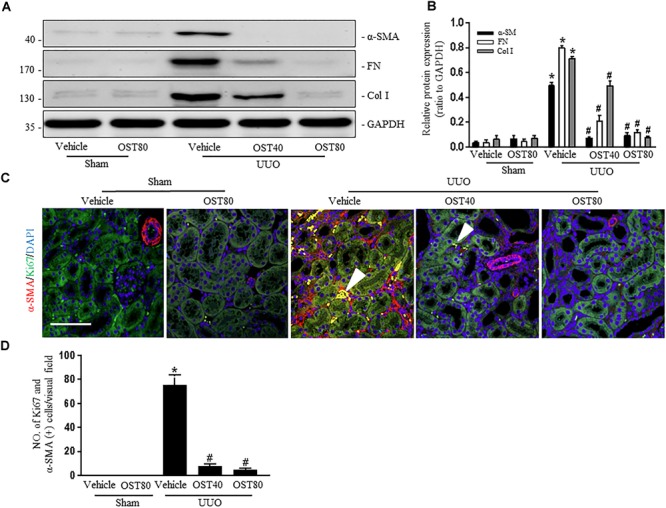
OST ameliorates renal interstitial fibrosis and myofibroblast proliferation in UUO-injured kidneys. Representative bands **(A)** and western blot analyses **(B)** for the expression of a-SMA, collagen I and fibronectin in the obstructed kidneys. **(C)** Representative double immunofluorescence staining of Ki67 (green) and α-SMA (red) and quantification **(D)** of the number of α-SMA- and Ki-67-(+) myofibroblasts per visual field on kidneys from the indicated groups (magnification of 200×). Scale bar = 100 μm. Nucleus was stained by DAPI. Arrowheads denote tubulointerstitial myofibroblasts with Ki67 and α-SMA -positive staining. Data are expressed as mean ± SEM. ^∗^*P* < 0.05 versus Sham + Vehicle; ^#^*P* < 0.05 versus UUO + Vehicle.

Quantification of MTC staining showed an 8.5% increase of collagen deposition in fibrosis in the cortex of obstructed kidneys from UUO mice compared to that from Sham-operated mice, which was considerably reduced by OST treatment (Figure 3A, quantification in Figure 3B). Immunostaining for FN, Col I, and α-SMA were carried out in kidney sections. Results show that OST treatment significantly attenuated ECM component (FN and Col I) deposition and α-SMA^+^ myofibroblast accumulation in obstructed kidneys from UUO mice (Figures 3A,B). Similar observations were confirmed by immunoblot analysis, in which the alteration of expression of α-SMA, FN and Col I were revealed in kidneys from UUO mice compared with control mice was significantly abolished by OST at the dose of 40 or 80 mg/kg/day (Figures 4A,B). Further, the effect of OST on renal myofibroblast proliferation was examined *in vivo*. Double immunostaining analysis indicated that UUO increased the number of α-SMA- and Ki-67-positive myofibroblasts in tubulointerstitial area by 75% compared with Sham group, while OST treatment resulted in a reduction in the number of Ki67 (+) proliferating interstitial myofibroblasts (Figures 4C,D). It is worth noting that mice in Sham+OST (80 mg/kg) did not develop any signs of distress such as weight loss, unkempt fur, or behavioral changes, suggesting that the dose used in our study did not cause overt toxicity.

### OST Regulates TGF-β1/Smad Signaling in TGF-β1-Induced NRK49F Cells and Mice With UUO Injury

**FIGURE 5:**
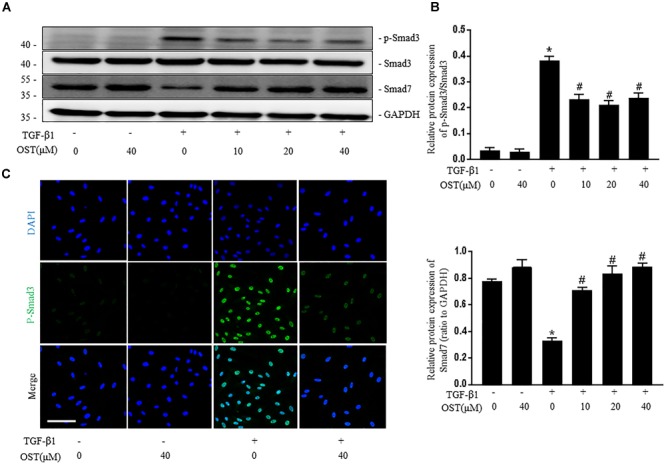
OST regulates TGF-β1/Smad signaling in NRK-49F cells. Representative bands **(A)** and Western blots analyses **(B)** of p-Smad3, Smad3, Smad7 in NRK-49F cells of different groups at 24 h after TGFβ1 (5 ng/ml) stimulation (magnification of 200×). Scale bar = 100 μm. **(C)** Representative micrographs of the immunofluorescence staining of p-Smad3. Data are expressed as mean ± SEM. ^∗^*P* < 0.05 versus untreated cells; ^#^*P* < 0.05 versus TGF-β1-stimulated cells.

**FIGURE 6:**
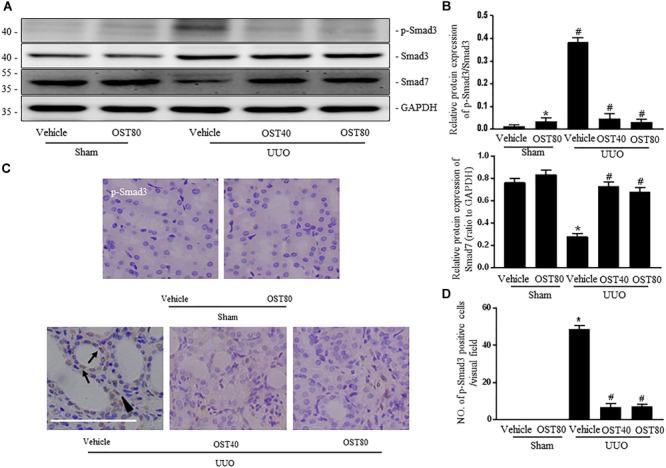
OST regulates TGF-β1/Smad signaling in obstructive kidneys of UUO mice. Representative bands **(A)** and Western blots analyses **(B)** of p-Smad3, Smad3, Smad7 in the obstructed kidneys at 1 week after UUO. **(C)** Representative micrographs of the immunostaining for p-smad3 in the kidneys from indicated group. Arrows indicate immunostaining positive tubular epithelial cells and arrowhead show immunostaining positive interstitial cells. Scale bar = 100 μm. **(D)** Quantification of the number of p-Smad3 positive cells per visual field on kidneys from the indicated groups. Data are expressed as mean ± SEM. ^∗^*P* < 0.05 versus Sham + Vehicle; ^#^*P* < 0.05 versus UUO + Vehicle.

TGF-β1 induced a robust phosphorylation of Smad3 and decreased expression of Smad7 in NRK-49F cells, while co-incubation with OST reduced p-Smad3 expression and maintained Smad7 expression in a dose-dependent manner (Figures 5A,B). Similarly, in the presence of OST, the abundance of p-Smad3 nuclear expression triggered by 5 ng/ml TGF-β1 was notably reduced (Figure 5C). The effect of OST on TGF-β1/Smad signaling in renal tissue was further explored. Mice challenged with UUO displayed increased expression of phosphorylation of Smad3 and decreased Smad7 expression, and OST treatment inhibited UUO-induced Smad7 loss and Smad3 phosphorylation (Figures 6A,B). Immunostaining showed that p-Smad3 was mainly expressed in the nuclear of renal tubular epithelial cells of UUO day 7 kidneys. Moreover, some cells distributed in the intertubular spaces are also immune-positive for p-Smad3 (Figure 6C). No significant immunolabeling is detected in the kidney from sham-operated and OST-treated animals (Figure 6D).

### OST Attenuates UUO-Induced EMT in Obstructive Kidneys

**FIGURE 7:**
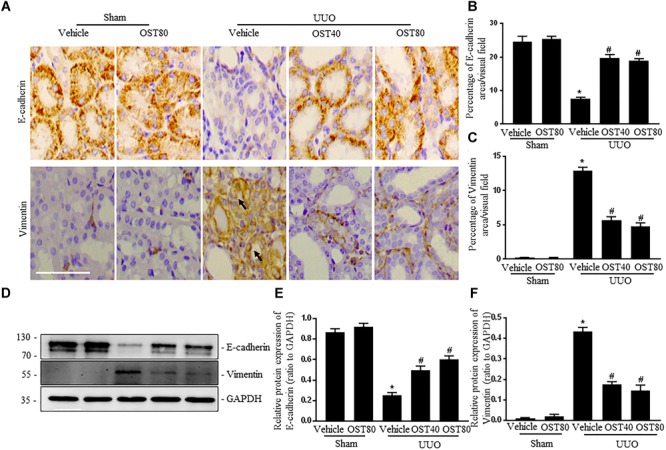
OST attenuates renal EMT in obstructive kidneys. Representative micrographs **(A)** and quantification **(B,C)** for E-cadherin and Vimentin in the obstructed kidneys from different groups as indicated. Arrows indicate immunostaining positive tubular epithelial cells. Scale bar = 100 μm. Representative bands **(D)** and western blots analyses **(E,F)** of E-cadherin and Vimentin in kidneys from the indicated groups. Data are expressed as mean ± SEM. ^∗^*P* < 0.05 versus Sham + Vehicle; ^#^*P* < 0.05 versus UUO + Vehicle.

**FIGURE 8:**
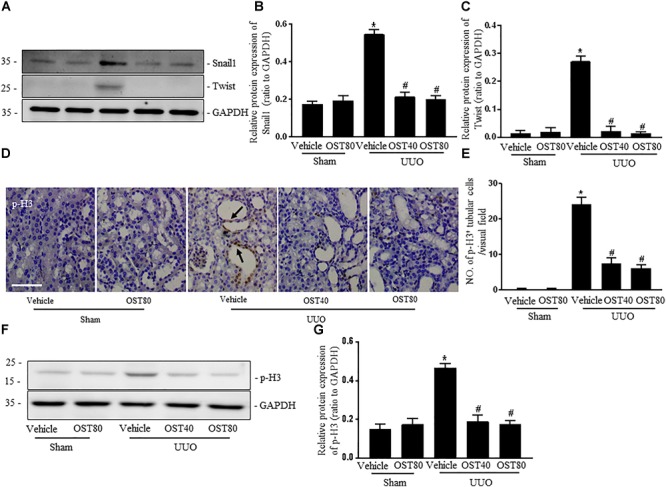
OST suppresses the expressions of Snail 1 and Twist and renal epithelial cell cycle arrest at the G2/M phase in obstructed kidneys. Representative bands **(A)** and western blots analyses **(B,C)** of Snail 1 and Twist in kidneys from the indicated groups. **(D)** Representative micrographs of immunostaining for phosphorylated serine 10 of histone H3 (p-H3, marker of G2/M cell cycle arrest) in different groups. Asterisk indicates the nuclear expression of p-H3. Scale bar = 100 μm. Arrows indicate immunostaining positive tubular epithelial cells. **(E)** Quantification of the number of p-H3 positive cells per visual field on kidneys from the indicated groups. Representative bands **(F)** and western blots analyses **(G)** of pH3 in the obstructed kidneys at 1 week after UUO. Data are expressed as mean ± SEM. ^∗^*P* < 0.05 versus Sham + Vehicle; ^#^*P* < 0.05 versus UUO + Vehicle.

Immunostaining as well as western blot analysis were used to determine development of EMT. As shown in Figures 7A–F, Mice undergoing UUO revealed decreased expression of E-cadherin and increased expression of Vimentin in the obstructive kidneys. Of note, Vimentin, the mesenchymal marker, was localized in extremely damaged renal tubular structures at the luminal and lateral side of the cell, which was consistent with the notion of tubular EMT in this model (Figure 7A). By contrast, OST treatment ameliorated these effects (Figures 7A–F). Enhanced expression of Snail1 and Twist, two transcriptional regulators of EMT, also corroborated the enrichment for EMT signature in fibrotic kidneys from UUO mice compared to control kidney and their expression was significantly downregulated in fibrotic kidneys of OST-treated mice (Figures 8A–C). G2/M phase arrest, a biologic consequence of EMT, was also investigated in fibrotic kidneys. Obstructive kidneys from UUO mice exhibited more p-H3^+^ tubular cells, as illustrated by the immunostaining, an effect that was significantly reduced after treatment with OST (Figures 8D,E). Immunoblot analysis also demonstrated an increased protein level of p-H3 in UUO mice, while OST treatment dramatically reduced it to the basal level (Figures 8F,G).

### OST Attenuates UUO-Induced Inflammation in Mice

**FIGURE 9:**
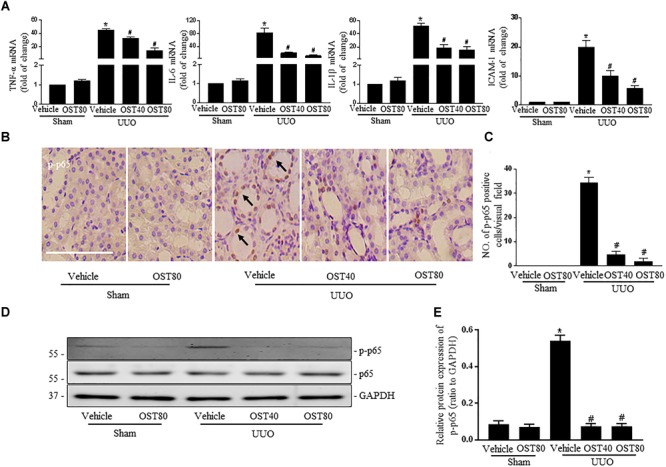
OST attenuates UUO-induced inflammation in mice. **(A)** Real-time PCR analyses for pro-inflammatory cytokines TNF-α, IL-6, IL-1β, and ICAM-1 in the kidney tissue among groups. **(B)** Representative image of p-p65 immunohistochemistry staining in kidneys. Arrows indicate the nuclear expression of p-p65. Scale bar = 100 μM. **(C)** Quantification of the number of p-p65 positive cells per visual field on kidneys from the indicated groups. Representative bands **(D)** and western blots analyses **(E)** of p-p65 in the obstructed kidneys at 1 week after UUO. Data are expressed as mean ± SEM. ^∗^*P* < 0.05 versus Sham + vehicle; ^#^*P* < 0.05 versus UUO + vehicle.

mRNA levels of pro-inflammatory cytokines, TNF-α, IL-6, IL-1β and ICAM-1 (Ricardo et al., 1996) were significantly upregulated in UUO kidneys compared to Sham, and OST treatment for 7 days attenuated these changes (Figure 9A). Moreover, there was a marked activation of NF-κB signaling in the obstructed kidneys after UUO, as evidenced by the increased number of phosphorylated NF-κB (p-p65) (Figures 9B,C) and increased ratio of p-p65 to total NF-κB (p65) and nuclear NF-κB (p65) translocation, which were ameliorated by OST treatment (Figures 9D,E).

### Statistical Analysis

Data were presented as means ± SEM values. The significance between the different groups was determined using One-Way ANOVA followed by Student–Newman–Keuls *post hoc* test. *P*-value < 0.05 was accepted as statistically significant.

## Discussion

The findings of present study show that (1) treatment with the coumarin derivative osthole (7-Methoxy-8-(3-methylbut-2-enyl)-2-chromenone) reduces fibroblast activation and proliferation *in vitro* and *in vivo*; (2) OST treatment blocks EMT program in injured kidney from UUO mouse; (3) OST administration suppresses activation of TGF-β1/Smad signaling pathway in fibroblasts and renal tissue (4) as well as inflammatory response in obstructed kidneys after UUO, which contribute to renal fibrosis. Overall, these data reveal that in addition to the anti-inflammation property of OST in various renal diseases (Zheng et al., 2013; Song W. et al., 2014; Huang and Dong, 2017), OST mitigates UUO-induced renal fibrosis mainly through reduction of fibroblast activation and EMT, at least in part, by inhibiting the TGF-β1/Smad signaling pathway.

OST, an ingredient commonly used in traditional Chinese herb, has been known for their potent anti-inflammatory and anti-tumor actions (Liao et al., 2010; Shokoohinia et al., 2018). Recently, studies reported that OST could diminish extracellular matrix deposition in hepatic and myocardial tissues (Chen et al., 2011; Liu et al., 2015). Consistently, TGF-β1-induced cardiac fibroblast activation and production of ECM components were blocked by OST (Liu et al., 2017). Given the conception that activation and proliferation of renal resident interstitial fibroblasts are the main events in the origin of myofibroblasts, the primary matrix-producing cells (Strutz and Zeisberg, 2006), cultured renal interstitial fibroblasts were exposed to TGF-β1, the central mediator of renal fibrosis, or serum, a mixture of multiple growth factors, to investigate the anti-activation and anti-proliferation role of OST. Our results clearly showed that OST inhibited TGF-β1-induced NRK-49F activation with reduced expression of α-SMA and production of ECM, fibronectin and type I collagen. In addition, OST suppressed proliferation of NRK-49F, as verified by the decreased the cell number and DNA synthesis rate stimulated by FBS. Cell proliferation is typically regulated by multiple cell cycle proteins, and the effects of OST in modulating cell cycle- and proliferation-associated genes have been demonstrated in previous studies (Jarzab et al., 2014; Wang et al., 2015; Hu et al., 2016; Li et al., 2017). In this study, OST prevented FBS-induced up-regulation of cyclin D1 (a key regulator of G1 to S transition) and PCNA (cell proliferation marker) as well as down-regulation of p21 Waf1/Cip1 (the negative regulatory factor of cell cycle progression) in FBS-induced NRK-49F. Collectively, our data indicated that OST attenuated phenotypic transformation and proliferation of fibroblasts *in vitro*.

*In vivo*, the effect of OST on renal interstitial fibrosis was investigated in mice challenged with UUO, an animal model featured by chronic renal injury and fibrosis in the tubulointerstitial compartment of the kidney. Our data revealed that OST (40 and 80 mg/kg/day) ameliorated fibrotic lesions with less collagen deposited in the renal interstitium and lower presence of α-SMA^+^ myofibroblasts. As in cultured cells, in obstructed kidneys from UUO mice, myofibroblast proliferation, measured by co-staining for Ki-67 and α-SMA, was inhibited after OST treatment. Together, the findings *in vitro and in vivo* showed that regulating activation and proliferation of renal fibroblasts may be an important therapeutic role for OST treatment in progressive renal fibrosis.

Smad signaling, also known as the canonical TGF-β pathway, is critically involved in renal fibrosis by inducing activation of myofibroblasts, transformation of tubular epithelial cells to myofibroblasts and tubular apoptosis (Sato et al., 2003; Zhang et al., 2015; Guo et al., 2018). In this pathway, TGFβ1 mainly transduces its fibrotic signal through activation of TGFβreceptor 1 (TGFβR1) followed by phosphorylation and nuclear translocation of Smad3, thus driving the progress of renal fibrosis. During fibrogenesis, Smad3 was generally overactivated, while Smad7, an inhibitory Smad has been shown to be degraded (Fukasawa et al., 2004). In the present study, our experiments reproduced this phenomenon in TGF-β1-stimulated NRK-49F cells as wells as UUO injury-induced kidneys and OST treatment reversed them. The *in vitro* modulatory effect of OST on TGFβ/Smad signaling was in line with previous study that OST increased Smad7 expression and decrease Smad3 expression in TGF-β1-treated cardiac fibroblasts (Liu et al., 2017), and may explain the decreased phenotypic transformation in TGF-β1-stimulated NRK49F when pre-incubation with OST. However, it should be noted that the non-Smad signaling, such as MAPK, AKT, and Wnt pathway, could also be the mechanism for OST in inhibiting kidney fibroblast activation. In addition, strategy targeting rebalancing TGF-β/Smad3/Smad7 signaling *in vivo* has been demonstrated to be effective for reducing renal fibrosis in obstructive nephropathy (Chung et al., 2013; Zhou et al., 2016). Thus, our data provide evidence that OST may act as a regulator of TGF-β/Smad signaling, thereby attenuates interstitial fibrosis in UUO kidney.

Epithelial-mesenchymal transition, a process defined as epithelial cells acquiring of mesenchymal features and motile phenotype, normally occurs in embryonic development, tissue fibrosis, and cancer (Kalluri and Weinberg, 2009). A complete EMT normally concurred with the phenomenon that the epithelial cells lose their polarity and cell–cell adhesions through downregulation of E-cadherin and gain the ability to migrate and invasion with a spindle-shaped mesenchymal morphology. It was recently reported that the injured renal epithelial cells undergoing a partial EMT process, without directly generating interstitial myofibroblasts, result in prolonged proliferation, cell cycle arrest, secretion of profibrotic factors and abnormal metabolic rearrangements, thus promoting fibrosis and parenchymal damage (Grande et al., 2015; Lovisa et al., 2016). The functional significance of EMT program in renal fibrosis was evidenced by the improved tubular health and a lower degree of interstitial fibrosis in the mice with two master regulators of EMT, Snail 1 or twist 1, was genetically deleted in proximal tubular cells, when they were challenged with different models of renal fibrosis (Lovisa et al., 2015). Vimentin, a member of the intermediated filament family of proteins as well as the marker of mesenchymal cells, has been demonstrated to be important in the development of EMT in renal tubular cells (Wang et al., 2018). Normally vimentin is not present in epithelial cells but re-expresses in injured or dedifferentiation tubular cells (Witzgall et al., 1994; Rastaldi et al., 2002; Tan et al., 2009; Kusaba et al., 2014). Previous studies in tumor cell lines had revealed that OST was able to reverse EMT program with inhibiting EMT transcription factor Snail or Twist expression. As oncogenic EMT and organic fibrosis share many of the same important signaling pathways and transcription factors that implicated in the development of EMT, it is likely that OST attenuated renal fibrosis through suppression of EMT (Lin et al., 2014; Feng et al., 2017). In support of this hypothesis, our current study showed that OST rescued UUO injury-induced EMT with increased expression of E-cadherin and decreased expression of Vimentin in tubular cells of kidneys, and concomitantly the less expression of Snail 1, Twist and p-H3. Herein, the results regarding cell arrest at G2/M phase in our study were inconsistent with previous studies where OST had been shown to induce G2/M arrest in tumor cells (Xu et al., 2011; Lin et al., 2017). It was reported that G2/M arrest associated-cell cycle protein p21 was differently regulated by EMT transcription factors, snail or twist in epithelial and tumor cell lines (Lovisa et al., 2015; Srivastava et al., 2016; Yang et al., 2017). Different cell type as well as the context of the diseases may explain some of this discrepancy. Altogether, our data provides evidence that the EMT-like program in renal tubular cells provoked by UUO surgery was attenuated after OST treatment. However, in this experiment setting, we did not directly address how these tubular cells that undergoing partial EMT engaged in the progress of renal fibrosis, which still needs to be further warranted.

The chronic inflammatory microenvironment characterized by up-regulation of inflammatory cytokines and growth factors are required for the development and progression of organ fibrosis (Misseri et al., 2004; Meng et al., 2014). It has been demonstrated that OST largely inhibited inflammation response in multiple experimental animal models, including chronic kidney disease (Huang and Dong, 2017). Our study echoed with these studies that OST exhibited an anti-inflammatory property by reducing mRNA expressions of the cytokines inflammatory cytokines such as TNF-α, IL-6, IL-1β, and ICAM. Additionally, OST suppressed proinflammatory transcription factor, NF-κB activation in renal tissue of UUO mice, which was not surprised in our context as previous studies has showed that OST could function as an inhibitor of NF-κB (Hua et al., 2013; Yang et al., 2014; Xie et al., 2015). Furthermore, NF-κB could induce transcription and stabilization of the Snail1 protein (Wu et al., 2009; Berzal et al., 2015). Thus, NF-κB/Snail as well as TGF-β/Smad3/Snail1 axis maybe the underlying mechanisms for the anti-EMT action of OST we demonstrated in this study.

## Conclusion

In summary, this study demonstrates that OST exhibites antifibrotic effects on obstructed nephropathy. The antifibrotic mechanisms of OST are associated with inhibition of fibroblast activation and proliferation, preservation of E-cadherin expression, and inactivation of the TGF-β/Smad and NF-κB signaling pathways. Despite the pleiotropic effects of OST on renal fibrosis indicated in our study, more detailed sets of investigation are warranted for the exact molecular basis and pharmacological action.

## Author Contributions

All authors have seen and approved the final version of the manuscript. SZ, JT, and EL conceived and designed the studies. SZ and QH performed most of the experiments with animal tissues and cultured cells, the statistical analysis, and contributed intellectually to the writing of the manuscript. XCai, SJ, NX, QZ, and XCao made the animal model and performed qPCR. SZ, MH, JT, and EL contributed to the conception of the article, the data interpretation, drafting of the manuscript, and revised the article for important intellectual content.

## Conflict of Interest Statement

The authors declare that the research was conducted in the absence of any commercial or financial relationships that could be construed as a potential conflict of interest.
